# An Improved Method for Surface Immobilisation of RNA: Application to Small Non-Coding RNA - mRNA Pairing

**DOI:** 10.1371/journal.pone.0079142

**Published:** 2013-11-14

**Authors:** Helen A. Vincent, Jack O. Phillips, Charlotte A. Henderson, Adam J. Roberts, Carlanne M. Stone, Charlotte E. Mardle, Louise E. Butt, Darren M. Gowers, Andrew R. Pickford, Anastasia J. Callaghan

**Affiliations:** School of Biological Sciences and Institute of Biomedical & Biomolecular Sciences, University of Portsmouth, Portsmouth, United Kingdom; University of Surrey, United Kingdom

## Abstract

Characterisation of RNA and its intermolecular interactions is increasing in importance as the inventory of known RNA functions continues to expand. RNA-RNA interactions are central to post-transcriptional gene regulation mechanisms in bacteria, and the interactions of bacterial small non-coding RNAs (sRNAs) with their mRNA targets are the subject of much current research. The technology of surface plasmon resonance (SPR) is an attractive approach to studying these interactions since it is highly sensitive, and allows interaction measurements to be recorded in real-time. Whilst a number of approaches exist to label RNAs for surface-immobilisation, the method documented here is simple, quick, efficient, and utilises the high-affinity streptavidin-biotin interaction. Specifically, we ligate a biotinylated nucleotide to the 3′ end of RNA using T4 RNA ligase. Although this is a previously recognised approach, we have optimised the method by our discovery that the incorporation of four or more adenine nucleotides at the 3′ end of the RNA (a poly-A-tail) is required in order to achieve high ligation efficiencies. We use this method within the context of investigating small non-coding RNA (sRNA) - mRNA interactions through the application of surface technologies, including quantitative SPR assays. We first focus on validating the method using the recently characterised *Escherichia coli* sRNA-mRNA pair, MicA-*ompA*, specifically demonstrating that the addition of the poly-A-tail to either RNA does not affect its subsequent binding interactions with partner molecules. We then apply this method to investigate the novel interactions of a *Vibrio cholerae* Qrr sRNA with partner mRNAs, *hapR* and *vca0939*; RNA-RNA pairings that are important in mediating pathogenic virulence. The calculated binding parameters allow insights to be drawn regarding sRNA-mRNA interaction mechanisms.

## Introduction

RNA is a multifaceted molecule with an ever-expanding repertoire of intra-, inter-molecular and ligand-binding functions. These include acting as the messenger for gene expression (mRNA), regulating gene expression (e.g. riboswitches, non-coding RNAs, siRNAs), catalysing biological processes (e.g. self-splicing ribozymes, the ribosome, telomerase) and providing mechanisms for viral infection [1], [2], [3], [4], [5]. Although RNA can act alone, most frequently its function requires interaction with proteins (e.g. RNA chaperones), other nucleic acids and/or small molecules. Access to simple tools for probing these interactions, specifically for the immobilisation (e.g. to sensor surfaces) and/or detection of RNA, is of increasing importance to the study of RNA.

The interactions of bacterial trans-acting small non-coding RNAs (sRNAs) with their mRNA targets are the subject of much current research. sRNAs, which range from 50–200 nucleotides, have become increasingly recognised as a novel and ubiquitous class of gene expression regulator [6]. They function through pairing with their mRNA targets via short regions of imperfect complementarity [7], in some cases promoted by the RNA chaperone protein, Hfq [8]. This sRNA-mRNA pairing has been shown to mediate either translational repression [9], [10] or translational activation [11]. Some sRNAs demonstrate a high specificity for a single mRNA target, whilst others are more promiscuous in their choice of binding partner [12]. An example of such sRNA variety is found in the *V. cholerae* quorum regulatory RNAs (Qrr sRNAs). Specifically, *V. cholerae* expresses four Qrr sRNAs that are thought to be functionally redundant, and that are involved in regulating mRNA expression of specific transcripts, thereby controlling biofilm formation and regulating pathogenic virulence [13], [14], [15]. When promoting infection (the low cell density phase), the Qrr sRNAs act as an ‘on’ switch to up-regulate *vca0939* mRNA by preventing the formation of a translation-inhibiting stem-loop structure within the 5′ UTR [14]. The *vca0939* gene encodes a GGDEF domain-containing protein that synthesises the intracellular signalling molecule cyclic di-GMP which is involved in controlling biofilm formation [14]. Concomitantly, the Qrr sRNAs act as an ‘off’ switch, down-regulating *hapR* expression, by pairing to the 5′ UTR, thereby blocking the ribosome binding site [14]. The HapR transcription factor represses the ToxR regulon which, in the absence of HapR, is activated and expresses the virulence factors cholera enterotoxin and the toxin-coregulated pilus [16]. Interestingly, the Qrr sRNAs in all *Vibrio* species have been shown to contain a conserved 21-nucleotide sequence predicted to imperfectly base-pair with the mRNA targets *hapR* and *vca0939* [15]. Determining the affinity of the interactions between such sRNAs and their mRNA targets is therefore important for furthering our molecular-level understanding of this important post-transcriptional gene regulation mechanism.

Sensor surface-based technologies such as surface plasmon resonance (SPR) and biolayer/dual polarisation interferometry are becoming increasingly popular for studying intermolecular interactions since both techniques offer sensitive detection in real-time. However, oriented immobilisation of one of the interacting partners in a manner that generates a homogenous surface, and allows observation of physiologically-relevant interactions, remains a challenge for most molecules, including RNA [17].

The main classes of immobilisation that are currently available are physical adsorption, covalent attachment and bioaffinity immobilisation [17]. Physical immobilisation relies upon ionic, electrostatic and/or hydrophobic interactions between the surface and the molecule to be immobilised (e.g. aminosilane surfaces) while covalent attachment is achieved through reaction of accessible functional groups with the surface (e.g. amine-coupling chemistry). Unfortunately, both physical and covalent strategies typically result in heterogeneous surfaces with randomly oriented molecules that are not ideal for interaction studies. Bioaffinity immobilisation (e.g. the streptavidin-biotin system) therefore has the advantage of allowing site-specific surface attachment to generate a homogeneous, oriented surface. The challenge then becomes how to efficiently incorporate the bioaffinity tag (biotin) into the molecule that is to be immobilised.

A number of approaches currently exist to incorporate bioaffinity tags, such as biotin, into RNA molecules. For example, RNA can be chemically synthesised to include the biotin label, but this approach is realistically limited to RNAs less than approximately 40 nucleotides in length due to the exponential decay in yield with increasing oligonucleotide length [18]. This limitation restricts subsequent interaction studies using tagged RNAs to short, minimal substrates containing known binding sites. In place of size-restrictive chemical synthesis, large biotin-labelled RNAs can be synthesised by *in vitro* transcription from a DNA template. For example, including a 5′-biotin-modified guanosine analogue in place of the usual GTP within the transcriptional mix allows T7 RNA polymerase to synthesise a 5′-biotin tagged RNA [19]. Alternatively, the 5′ end of the RNA can be chemically modified to incorporate a sulfhydryl group, which in turn can react with a haloacetyl-activated biotinylation reagent [20]. Nonetheless, attachment of the RNA to the surface via the 5′ end may not be suitable when studying interactions involving, or in the vicinity of, the 5′ end of the RNA. Therefore, methods to add a biotin tag to the 3′ end of an RNA molecule would be favourable in such circumstances. For instance, periodate chemistry can be used to convert the 3′ terminal ribose to a dialdehyde; subsequent reaction with biotin-hydrazine yields a 3′-biotinylated RNA molecule [21]. However, since RNA degradation is a constant threat to the researcher, the requirement to subject RNA to multiple, extended, chemical steps may be undesirable. Instead, enzymatic approaches can be used. Such approaches include that of T4 DNA ligase-mediated splinted ligation of two RNA molecules, one of which incorporates a biotin-tag, which are hybridised onto a DNA carrier [22]. Unfortunately, the requirement of forming the critical ligation-competent complex, in which the two RNA molecules to be ligated are annealed to the DNA splint, reduces the efficiency of this method. Another, more straightforward, enzymatic approach to 3′-end labelling involves T4 RNA ligase being used to directly ligate a 5′-adenosyl-pyrophosphate biotin-conjugate to an RNA molecule [23], [24], and this is the approach we have explored further (see below).

With the aim of devising an SPR study to explore sRNA-mRNA interactions, we undertook biotin-tagging of RNA molecules for surface immobilisation using the approach of ligating a biotinylated nucleotide to the 3′ end of the RNA using T4 RNA ligase. Crucially, we discovered that the presence of multiple adenine nucleotides at the 3′ end of the RNA was essential for high-efficiency ligation. This requirement was demonstrated for RNAs of a range of sizes, including substrates up to several hundred nucleotides in length. Validation that our optimised method did not impact RNA function was achieved using MicA, an sRNA produced in *E. coli* in response to cellular stress. MicA’s interaction with its target transcript, *ompA*, has been well characterised with a low nanomolar equilibrium dissociation constant (K_D_) previously determined by electrophoretic mobility shift assay (EMSA) analysis [25], [26], [27]. We were similarly able to obtain low nanomolar affinities for the MicA-*ompA* interaction when monitoring binding of either MicA or *ompA* to its immobilised biotin-tagged partner RNA by SPR. With the methodology validated, we have demonstrated the use of this approach to explore the interaction of a *V. cholerae* Qrr sRNA with its mRNA targets.

## Materials and Methods

### RNA Synthesis

RNAs, excluding *rpoS* constructs, (Table S1 in File S1), with both native sequence and incorporating 3′ poly-A-tails, were synthesised by *in vitro* transcription using a MegaScript T7 kit (Life Technologies) from PCR templates generated by gene synthesis from overlapping primers [28]. Each sequence was designed to contain a T7 promoter sequence (5′-TAATACGACTCACTATA) and up to 3 guanines at the 5′ end to enhance the yield from transcription. For *rpoS* constructs (Table S1 in File S1), the plasmid *rpoS*-Blunt II TOPO (encoding -576 to +10 of *rpoS* with a 5′ T7 promoter sequence) was used as template DNA. For RNAs incorporating 3′ poly-A-tails, between four and eight adenines were appended to the native sequence with, in each case, the extension length being limited by the need to maintain the native structure of the RNA as determined by MFold analysis [29]. The DNA primer sequences used to prepare the native and 3′ poly-A-tail RNAs are provided in Table S2 in File S1. The transcribed RNAs were purified prior to ligation using a MegaClear kit (Life Technologies).

### Ligating U-biotin to RNA

Reaction mixtures contained 5 µM RNA, 10% (v/v) DMSO, 5 µM U-biotin (uridine 5′,3′-(bis)phosphate with biotin linked through the 3′ phosphate via an extended organic linker; Fig. S1 in File S1; Dharmacon), 100 units of T4 RNA ligase 1 (NEB) in 50 mM Tris-HCl pH 7.8, 10 mM MgCl_2_, 5 mM DTT and 1 mM ATP. Reactions were incubated at 37°C for 60 min. To achieve high ligation efficiencies, the RNA had a poly-A-tail of four to eight adenine nucleotides appended to the 3′ end (as described above in ‘RNA Synthesis’). Subsequent separation of the RNA and RNA-biotin conjugate from the other reaction components was achieved by two cycles through a Micro Bio-Spin 6 size exclusion column (Bio-Rad). Chromatographic clean up was confirmed using an analytical gel filtration column (Zorbax 450, Agilent) on a liquid chromatography system (Dionex).

### Polyacrylamide Gel Electrophoresis (PAGE) and Electro-blotting

Ligation reactions were analysed on 20% (w/v) polyacrylamide (19∶1 acrylamide:bis-acrylamide) denaturing-gels containing 7 M urea. Gels were stained with SYBR-Gold and visualised using a UV transilluminator. RNA bands were quantified using Gene Tools software (SynGene). These data were used to determine the yield of biotin-labelled RNA, calculated as a percentage of the total RNA present. To confirm that U-biotin had been ligated to the RNA, the RNA was transferred to a Nylon+ (Bio-Rad) membrane by electro-blotting in 0.5×TBE, and cross-linked to the membrane using UV light. The membrane was probed with streptavidin-horseradish peroxidise conjugate, and detected with enhanced chemiluminescence.

### Surface Immobilisation to Microarray Slides

Aliquots (10 µL) of 1 mg/mL streptavidin in phosphate-buffered saline pH 7.4 (PBS) were pipetted onto NHS-activated slides (Nexterion H, Schott), incubated for 30 min at 37°C in a humidified chamber, washed three times with PBS containing 0.05% (v/v) Tween (PBS-T), twice with water, and then air-dried. The slide was incubated in 50 mM ethanolamine at room temperature for 30 min prior to washing, as described above. Once air-dried, 10 µL of 400 nM test sample (biotin-tagged RNA) and control samples (non-biotin-tagged RNA, biotin-tagged control RNA and buffer) were pipetted onto the streptavidin, and incubated for 30 min at 37°C in a humidified chamber, washed three times with PBS and then air-dried. The slide was blocked with 200 nM bulk mRNA (Sigma) for 30 min at 37°C in a humidified chamber prior to washing as above. The slide was then incubated overnight at room temperature with 400 nM Cy-labelled probe RNA in PBS, washed three times with PBS and air-dried. The slide was imaged at 550 nm excitation using a slide scanner (QScan, Genetix).

### SPR Interaction Studies

On-chip RNA immobilisation on the test flow cell was achieved by injecting 10 nM biotin-tagged RNA in HEPES-buffered saline pH 7.4 (HBS; GE Healthcare) over a streptavidin-coated sensor chip (GE Healthcare) at 10 µL/min until ∼200 RU (which equates to ∼200 pg/mm^2^ or, depending on the size of the RNA, ∼1–10 fmol/mm^2^, calculated using the standard manufacturer’s conversion of 1 RU = 1 pg/mm^2^) of sample were immobilised. The same procedure was used to immobilise biotin-tagged control molecules on the control flow cell. The blank flow cell was left untreated. To monitor interactions with binding partner RNA molecules, single-cycle kinetic experiments were conducted. This involved consecutive 2 min injections of 5 different concentrations of binding partner molecule (in either the 0–0.25 µM or 0–10 µM range), each separated by a 1 min dissociation phase and a final dissociation of ∼8 min. The experiment was run at 60–90 µL/min in HBS buffer using a T100 Biacore instrument (GE Healthcare) and the single cycle kinetic method within the Biacore control software. Data were analysed using T100 BiaEvaluation software (GE Healthcare) with curve fitting to the data achieved using a 1∶1 binding model.

## Results

### Improving the Yields of Biotin-labelled RNA

**Figure 1:**
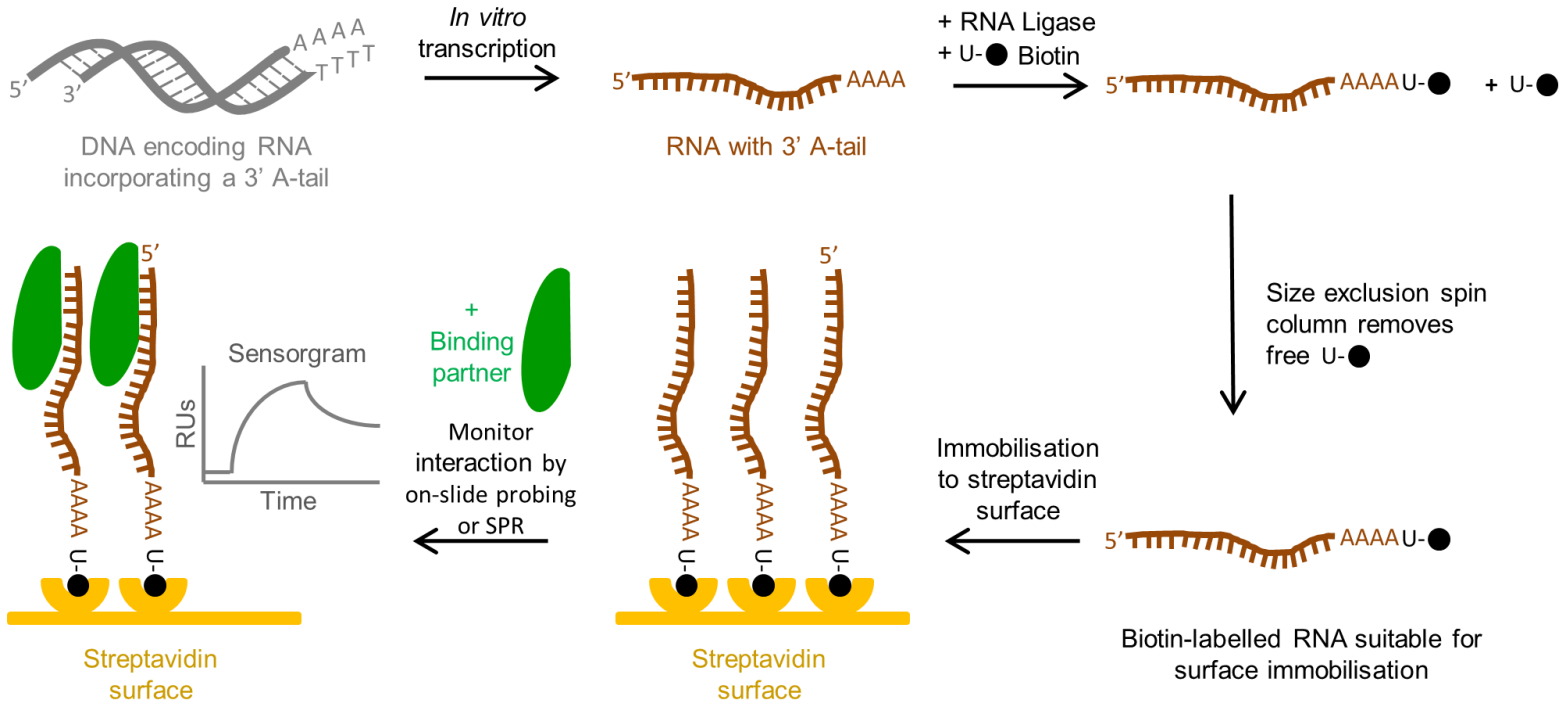
Schematic of the strategy for surface immobilisation of large RNAs. RNA (brown) is prepared by *in vitro* transcription and terminated by template run-off, from the corresponding DNA template (grey); the RNA incorporated between four and eight adenines at the 3′ end. A biotinylated uridine (U-biotin, filled black circle) is then ligated onto the 3′ poly-A-tail RNA by T4 RNA ligase. Excess U-biotin was then removed from the labelled RNA using a size exclusion spin column leaving it ready for sensor surface immobilisation. Following immobilisation of the biotin-labelled RNA to a streptavidin sensor surface (yellow), interactions of binding partner molecules (green) with the immobilised RNA can then be undertaken by on-slide probing or SPR analysis. A schematic sensorgram illustrative of a binding event for SPR immobilisation is shown.

The process of attaching a biotin molecule to the 3′-end of large RNAs, and subsequent immobilisation of the biotin-labelled RNA onto a sensor surface for interaction studies, is shown schematically in Figure 1. Briefly, RNA was prepared by *in vitro* transcription to generate an RNA with between four and eight adenines at the 3′ end. The number of adenines incorporated was controlled by the DNA template sequence used, with between four and eight adenines appended to the native sequence. In each case, the adenine extension length was limited by the need to maintain the native structure of the RNA as determined by MFold analysis [29]. A biotinylated uridine (U-biotin; Figure S1 in File S1) was then ligated onto the 3′ A-tail RNA by T4 RNA ligase. Excess U-biotin was then removed from the labelled RNA using a size exclusion spin column, leaving it ready for sensor surface immobilisation and subsequent SPR analysis with potential binding partner molecules.

**Figure 2:**
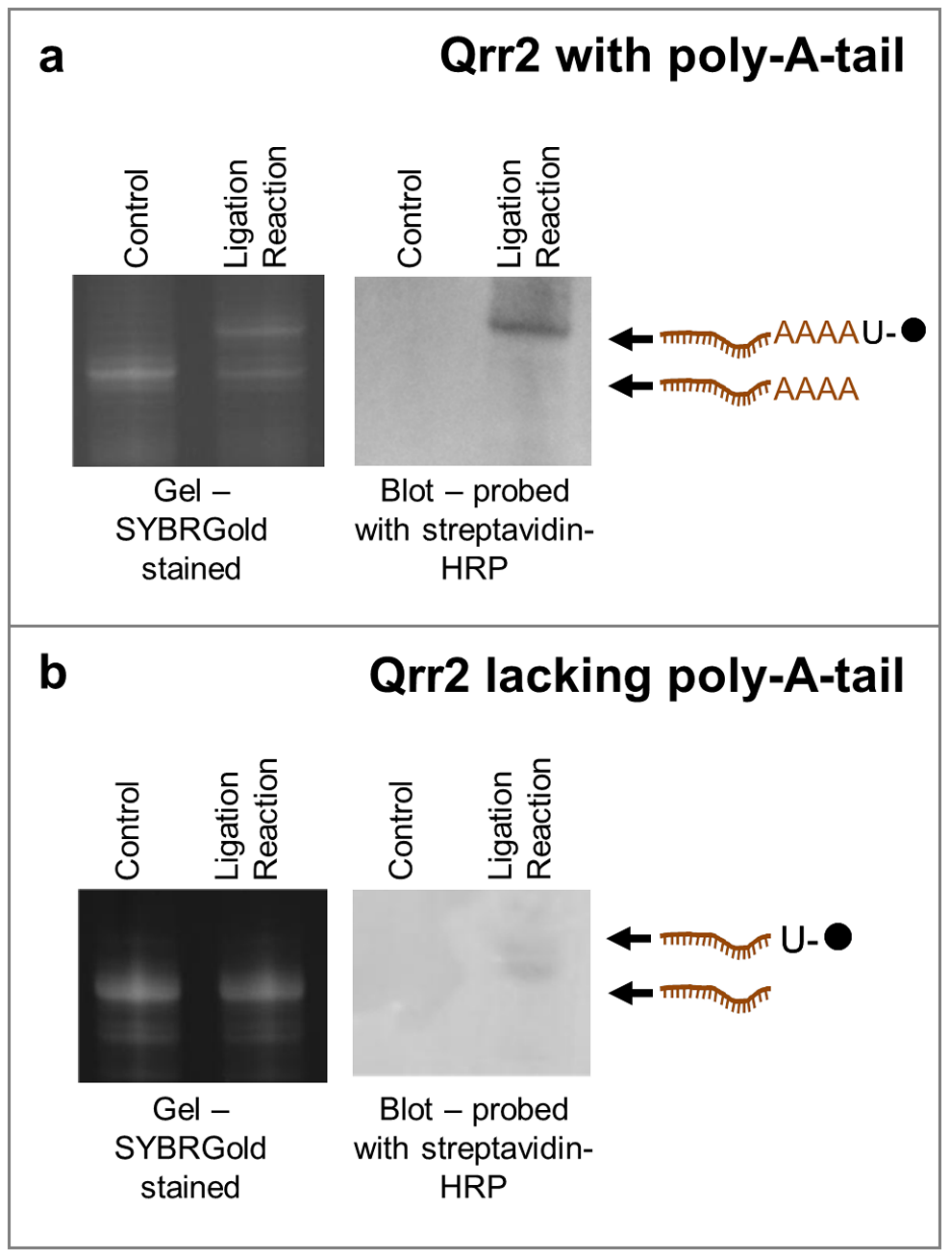
Analysis of the ligation reaction for Qrr2 sRNA (a) with and (b) without A-tails. Gels were stained with the SYBR-Gold, whereas blots were probed with streptavidin-HRP to detect biotin-labelled RNA. Schematic representations of RNA species identified on the gels/blots are shown. The sequences of the RNAs are given in Table S1 in File S1.

Figure 2, and Figures S2 and S3 in File S1, show the results of typical ligation reactions analysed by denaturing PAGE. Biotin-labelled RNA migrates more slowly through the gel relative to the starting RNA molecule, allowing yield determination (Table 1). Subsequent blotting followed by detection with a streptavidin-horseradish peroxidase conjugate confirmed the presence of biotin in the reaction product (Figure 2 and Figures S2 and S3 in File S1). Efficient removal of excess U-biotin following the ligation reaction is shown in Figure S4 in File S1, and sensor surface immobilisation is demonstrated by binding of the biotin-labelled RNA to a streptavidin coated SPR sensor chip (Figure S5 in File S1).

**Table 1:** Ligation yields for U-biotin to RNAs with A-tails. RNA sequences are given in Table S1 in File S1.

RNA	sRNAQrr1	sRNAQrr2	sRNAQrr3	sRNAQrr4	*hapR*mRNA	*vca0939*mRNA	*rpoS* mRNA*(*short)	sRNAMicA	*ompA*mRNA	*rpoS* mRNA(long)	sRNADsrA
**Nucleotide** **length**	99	110	110	110	101	61	413	79	161	592	94
**% Yield**	94	61	67	76	60	62	55	63	53	53	49

The important step for obtaining high ligation efficiencies with this method is the presence of four to eight adenine nucleotides at the 3′ end of the RNA to be biotin-labelled. Indeed, preliminary data indicated adenine to be the preferred 3′ end nucleotide (Figure S6 in File S1) and an A-tail of at least 3 adenines was required for ligation enhancement (Figure S7 in File S1). In the absence of this ‘poly-A-tail’, ligation trials resulted in little or no biotin-labelling of the RNA molecule of interest (Figure 2 and Figure S2 in File S1), consistent with reports in the literature for ligations with long structured RNAs with varying 3′ end nucleotide composition [30]. Studies on the substrate specificity of T4 RNA ligase indicated that the nature of the 3′ end of the acceptor RNA is important for ligation efficiency [31], [32]. The simple inclusion of a poly-A-tail of an appropriate length at the 3′ end of our RNAs (see Table S1 in File S1 for the RNA sequences) dramatically improved ligation efficiency to between 49% and 94% (Table 1).

The efficiency of ligating a longer biotinylated oligoribonucleotide (U_5_-biotin) onto a poly-A-tailed RNA was also tested, and the results of two typical ligation reactions are shown in Figure S8 in File S1. Yields were typically lower than when labelling the same RNA with U-biotin (34% on average for U_5_-biotin compared to 63% on average for U-biotin; Table 1 and Table S3 in File S1). Nevertheless, these yields were again a dramatic improvement when compared to attempts to biotin-tag RNAs lacking the poly-A-tail (Figure S8 in File S1). This demonstrates that the addition of adenines to the 3′ end of the RNA is essential for obtaining high ligation efficiencies, thereby making immobilisation of large RNAs (≥60 nucleotides in length) to a sensor surface, for subsequent analysis of interactions with binding partners, a realistic possibility.

We have demonstrated that our strategy can be used to efficiently label eleven different RNA molecules that range in length from 61 to 592 nucleotides, suggesting that the approach could prove to be applicable for any RNA. The method is straightforward, fast (∼90 minutes when starting from poly-A-tailed RNA) and requires only commercially-available reagents and equipment (T4 RNA ligase, U-biotin, spin columns) in addition to the RNA to be biotin-tagged. The poly-A-tailed RNA can be readily prepared by including the adenines in the *in vitro* transcription template, as described here. Whilst we demonstrate the method for larger, transcribed RNAs (≥60 nucleotides in length), it could be equally applicable to shorter chemically synthesised RNA molecules incorporating a poly-A-tail. However, if the method is to be applied to such chemically synthesised RNAs, then the 5′ end should not be a monophosphate as that would allow undesired circularisation and/or concatenation of the RNA in the presence of RNA ligase.

### Biotin-labelled RNA is Surface-immobilised in an Active Form

**Figure 3:**
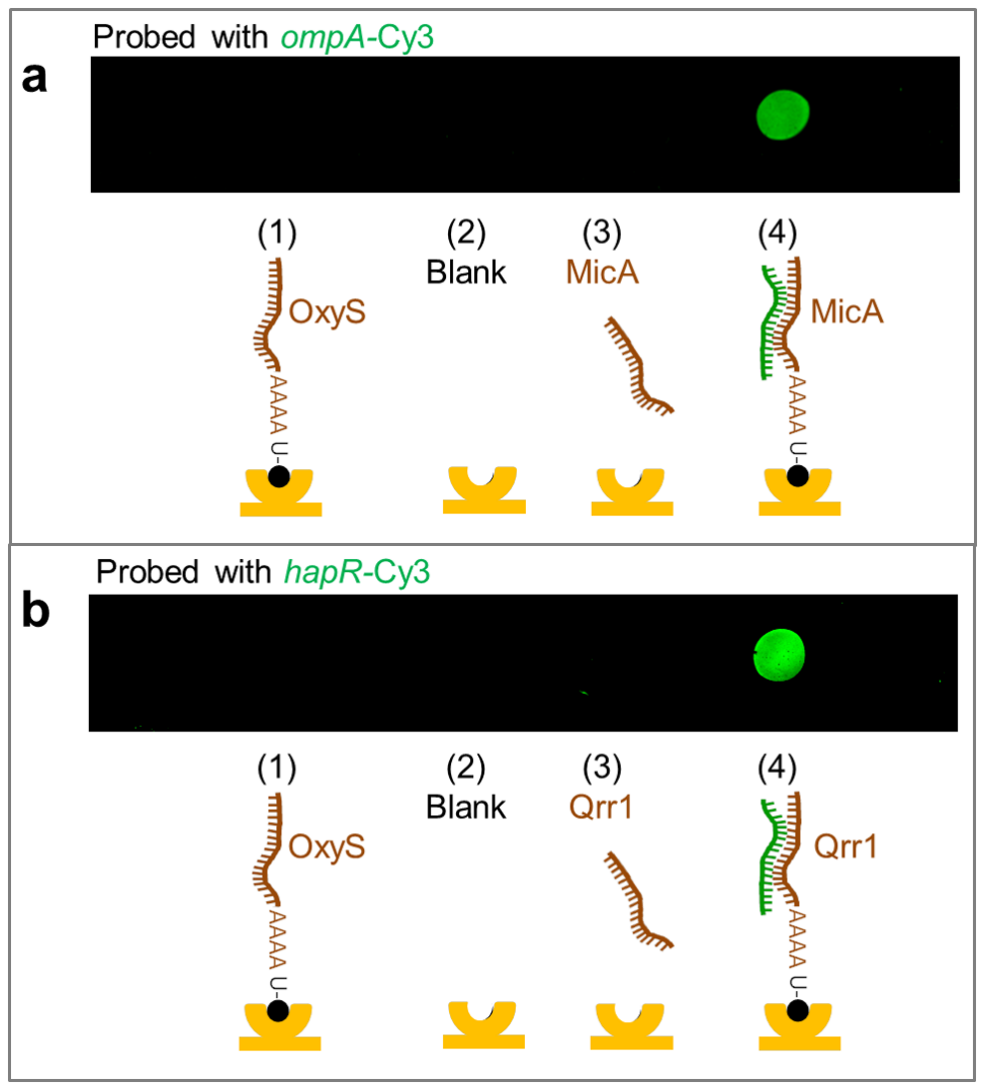
Probing of surface-immobilised biotinylated-sRNA with partner mRNA-Cy3. (a) Streptavidin-coated microarray slide with control spots of (1) biotin-OxyS, (2) blank surface, (3) sRNA MicA, and test spot of (4) biotin-MicA. The surface was probed with Cy3-labelled *ompA*. The specific *ompA i*nteraction with surface-immobilised biotin-MicA is shown by the green spot. (b) As for (a) but in this case the test spot (4) is biotin-Qrr1 and the control sRNA spot (3) is Qrr1. The surface was probed with Cy3-labelled *hapR*. The specific *hapR* interaction with surface-immobilised biotin-Qrr1 is seen by the green spot. Schematic illustrations of the interactions occurring in (a) and (b) are shown beneath the microarray slides with the streptavidin surface in yellow, sRNAs in brown and Cy3-labelled mRNA in green.

The ability to simply and efficiently biotin-label large RNAs (≥60 nucleotides in length) has allowed us to apply our method to the study of biologically relevant RNA-RNA interactions using surface technologies. Here, we examine the binding of the *E. coli* sRNA, MicA, to its mRNA target, the 5′ UTR of *ompA*. This interaction has been well characterised, with the affinity of MicA-*ompA* identified to be in the low nanomolar range [25], [26], [27]. Biotin-labelled MicA was immobilised to the surface of a streptavidin-coated slide and probed with Cy3-labelled *ompA* (Figure 3a, position 4). Two control RNAs were also tested for *ompA* binding. The first was MicA without a biotin tag (Figure 3a, position 3), and the second was another biotinylated-sRNA, OxyS, involved in the regulation of alternative mRNA targets *in vivo* [33] (Figure 3a, position 1). Specific binding of *ompA* to immobilised MicA-biotin was seen. This demonstrated that MicA, incorporating a poly-A-tail and biotin-tagged at the 3′ end, can be surface-immobilised in an active form. The same method was then used to assess whether other sRNAs could be similarly surface-immobilised in an active form. Specifically, a *V. cholerae* Qrr sRNA (Qrr1), known to interact with the 5′ UTR of its mRNA target, *hapR* [13], [14], was tested. The Qrr sRNA incorporating a poly-A-tail was biotin-tagged at the 3′ end, and then immobilised to a streptavidin-coated slide (Figure 3b, position 4). Appropriate controls of non-biotin-tagged Qrr sRNA (Figure 3b, position 3) and a biotin-tagged control sRNA, OxyS, (Figure 3b, position 1) were included. After probing with Cy3-labelled *hapR*, only specific binding to the biotin-tagged Qrr sRNA was seen (Figure 3b, position 4). These results confirm that sRNAs, biotin-labelled using our method, can be immobilised in an active form as they remain capable of binding to their cognate partner mRNAs.

### Quantitative Study of Biotin-labelled RNA Interactions with Binding Partner RNAs by SPR

**Figure 4:**
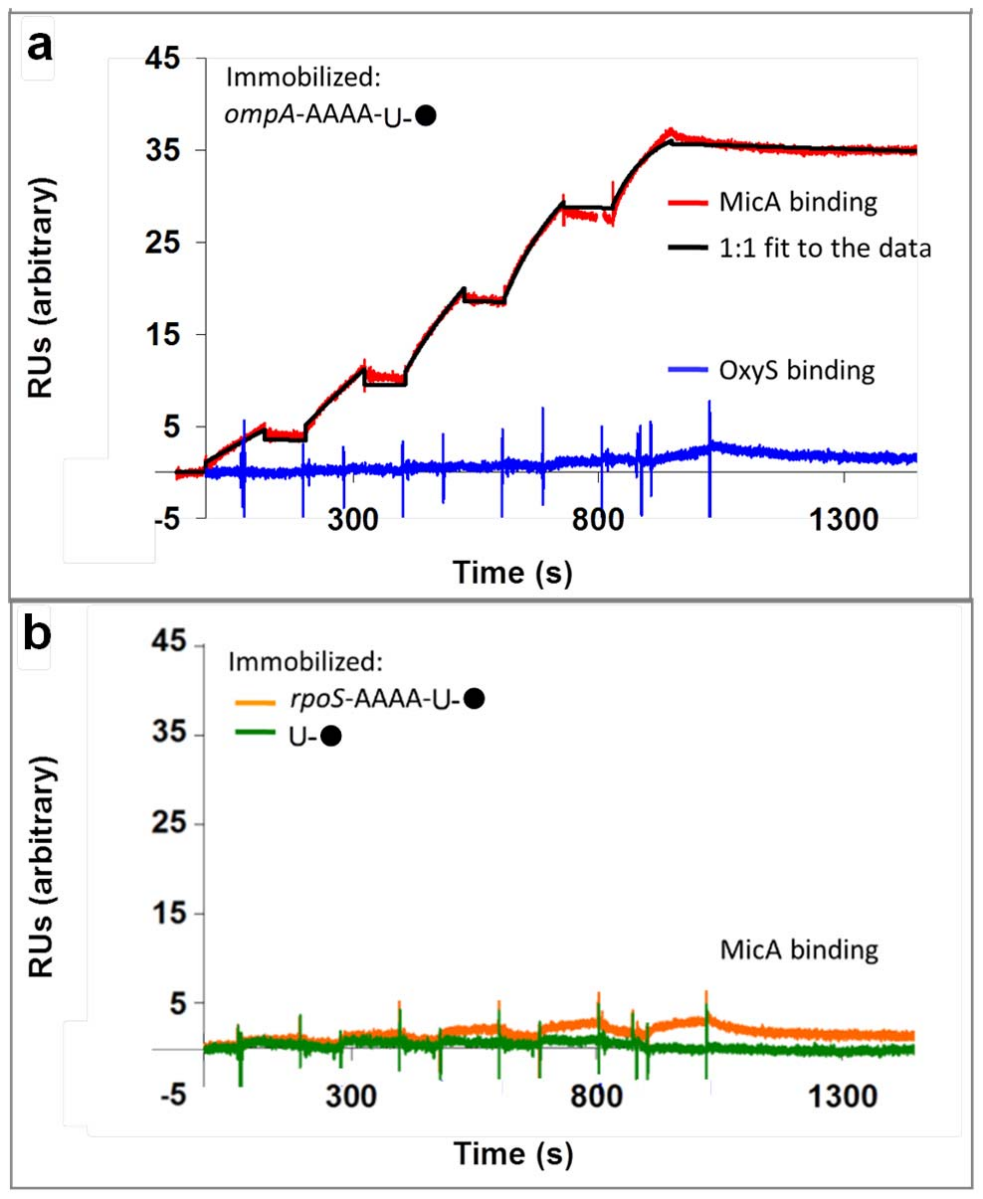
SPR analysis of RNA-RNA interactions. (a) Surface-immobilised biotin-*ompA*. Example sensorgrams of sequential injections of MicA (red) or OxyS (blue) from 0–10 µM; MicA data fit (black) with chi^2^ = 0.20 RU^2^. (b) Control sensorgrams of sequential injections of MicA from 0–10 µM over surface-immobilised *rpoS* mRNA (orange) or U-biotin reagent (green).

Having demonstrated that RNAs can be tagged and surface-immobilised in an active form on a microarray slide, a quantitative approach was taken to explore sRNA-mRNA interactions using SPR. The 5′ UTR of the mRNA *ompA* was biotin-labelled (Table 1 and Table S1 in File S1) and surface-immobilised to a streptavidin-coated SPR sensor chip. Kinetic analysis of its interactions with the sRNA target MicA was conducted (Figure 4a, red line). The binding data collected were fit to a 1∶1 binding model which identified the on-rate, off-rate and K_D_ for the interaction (Figure 4a, black line; Table 2). Similar binding data were also identified for the reverse experiment in which biotin-labelled sRNA MicA (Tables 1 and Table S1 in File S1) was immobilised to the streptavidin sensor surface and *ompA* (without a poly-A-tail) was tested as the binding partner (Table 2). The close agreement between the kinetic values determined for these experiments demonstrates that the presence of the A-tail does not significantly impact on the functional interaction of the RNAs, and that the interaction is the same irrespective of which molecule is immobilised. Indeed, the presence of the poly-A-tail may provide a positive effect on the surface-immobilised RNA by acting as a linker, thereby ensuring that the immobilised RNA is not sterically-hindered by the surface to which it is tethered, such that it is unable to interact with its binding partner molecule. We further demonstrated the observed MicA-*ompA* interaction to be specific, since a control sRNA (OxyS, which does not target the mRNA *ompA in vivo* [33]) did not bind to immobilised *ompA* (Figure 4a, blue line). Similarly, no interaction could be detected between MicA and immobilised U-biotin control reagent (Figure 4b, green line) or a control mRNA, *rpoS*, which is not a target of MicA *in vivo* [34] (Figure 4b, orange line).

**Table 2:** Kinetic data for sRNA-mRNA interactions from SPR analyses.

Surface-immobilised RNA	Analyte RNA	k_on_ (M^−1^s^−1^)	k_off_ (s^−1^)	K_D_ (nM)
*ompA*	MicA	(1.3+/−0.1)×10^3^	(5.0+/−1.1)×10^−5^	38+/−8.0
MicA	*ompA*	(1.7+/−0.7)×10^3^	(4.9+/−1.6)×10^−5^	28+/−3.5
*hapR*	Qrr3	(1.4+/−0.6)×10^4^	(1.4+/−0.7)×10^−4^	11+/−4.3
*vca0939*	Qrr3	(6.2+/−2.2)×10^3^	(3.6+/−1.9)×10^−4^	57+/−11

**Figure 5:**
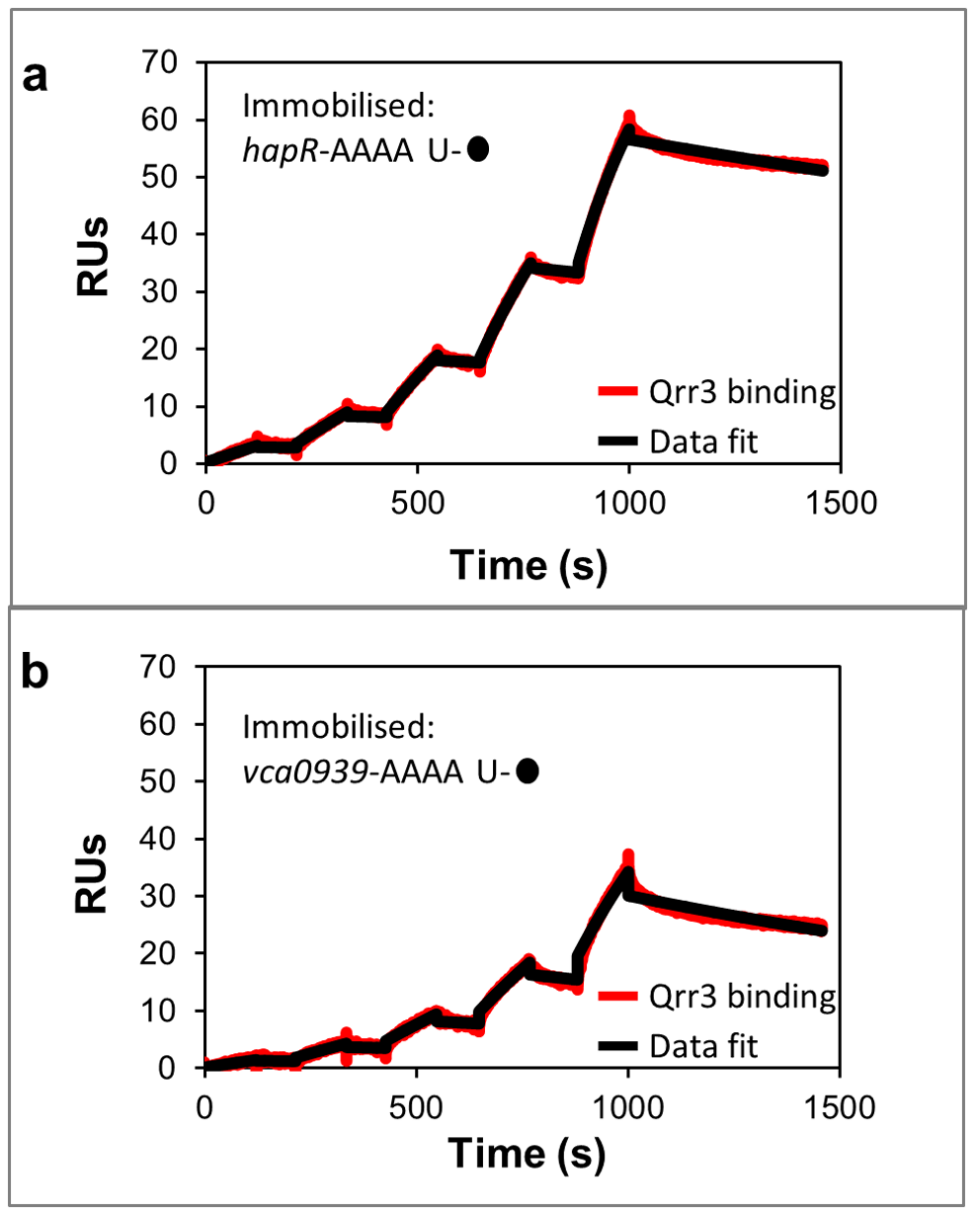
SPR analysis of Qrr3-mRNA interactions. (a) Surface-immobilised biotin-*hapR*. Example sensorgram of sequential injections of Qrr3 from 0–0.25 µM; data fit (black) with chi^2^ = 0.28 RU^2^. (b) Surface-immobilised biotin-*vca0939*. Example sensorgram of sequential injections of Qrr3 from 0–0.25 µM; data fit (black) with chi^2^ = 0.41 RU^2^.

We next characterised the interactions of a *V. cholerae* Qrr sRNA, namely Qrr3, with its mRNA binding partners *hapR* and *vca0939* [13], [14]. Recent studies using EMSAs have suggested the functionally redundant Qrr sRNAs bind to *hapR* with K_D_s in the ∼250–375 nM range [15]. For comparison, SPR was used to determine the binding affinity of the Qrr3 sRNA to *hapR*. Specifically, biotin-tagged 5′ UTR RNA of *hapR* was immobilised to a streptavidin-coated SPR sensor chip and kinetic analyses of the interactions with Qrr3 sRNA were tested. The binding data collected were fit to a 1∶1 binding model, and the on-rate, off-rate and K_D_ were calculated. The results suggest that a stable sRNA-mRNA complex is formed (Figure 5a and Table 2). However, predicted base-pairing between the mRNA target *hapR* and Qrr sRNAs, such as Qrr3, suggests that the strength and extent of pairing is slightly altered in comparison to that identified for the Qrr-*vca0939* pairing [14]. Consequently, a marginally different interaction affinity may be anticipated for the latter interaction when compared to the former. To investigate this, an analogous experiment to that conducted for the Qrr3-*hapR* interaction was carried out to determine the on-rate, off-rate and K_D_ of the Qrr3-*vca0939* pairing (Figure 5b and Table 2). A slower on-rate is identified for the Qrr3-*vca0939* interaction compared to that determined for Qrr3-*hapR* whilst the off-rates for both complexes are seen to be comparable. Collectively, this yields a slightly weaker K_D_ for the Qrr3-*vca0939* interaction, compared to that observed for the Qrr3-*hapR* interaction.

## Discussion

We have described a simple, quick and efficient strategy to biotinylate the 3′ end of biologically relevant RNAs, ranging in length from 61 to 592 nucleotides, in order to facilitate their immobilisation to a sensor surface. Crucial to achieving high levels of biotin-tagged RNA, we identified that a tail of four to eight adenine nucleotides were required at the 3′ end of the RNAs in order to support the efficient T4 RNA ligase-mediated addition of a biotinylated nucleotide. It is possible that the poly-A-tail improves access of the RNA chain to the ligase active site. Most likely, the improved ligation efficiency is a consequence of the substrate specificity of T4 RNA ligase as the enzyme requires a single stranded 3′ terminus and is sensitive to the nature of the 3′ end of the acceptor RNA [31], [32]. Consistent with these earlier findings, our data shows that a single stranded poly-A-tail appears to be preferred over poly-C, -G, or -U tails for enhancing the 3′ biotin-ligation efficiency of the structured RNAs tested here (Figure S6 in File S1). This indicates the additional importance of the nature of the nucleotides comprising the single stranded 3′ tail in supporting enhanced yields. This allowed us to modify a recognised - albeit currently not highly used – approach [23], [24], thereby making it more user-friendly and time-efficient. We have further shown that 3′ biotin-tagging of the RNA has no detrimental impact on RNA function when used to facilitate surface-immobilisation, since specific pairing of biotin-tagged RNAs with cognate partner RNAs was observed. Indeed, the incorporation of the poly-A-tail to the surface-immobilised RNAs likely facilitates binding by acting as a steric spacer, thereby precluding any potential steric issues with the immobilisation-surface. With the approach validated, we have exploited the method in the quantitative analysis of biologically-relevant sRNA-mRNA interactions on a sensor surface; something which has previously been challenging due to the lack of simple immobilisation strategies for structured, large RNAs (≥60 nucleotides in length).

SPR analysis of the MicA-*ompA* interaction identified similar on-rates, off-rates and K_D_s, irrespective of whether MicA or *ompA* were immobilised or used as the analyte in the experiment (Table 2). On-rate values lower than 10^5^ M^−1^ s^−1^ are generally accepted as illustrating slow complex formation [35]. Thus the MicA-*ompA* association was seen to be fairly slow (Table 2). This is not unexpected since the RNA chaperone protein, Hfq, is known to facilitate sRNA-mRNA pairing *in vivo* [8]. However, with off-rates below 10^−4^ s^−1^ considered as illustrating slow complex dissociation [35], it was seen that, once paired, the MicA-*ompA* dissociation was slow and thereby demonstrated MicA-*ompA* to form a stable complex. Low nanomolar K_D_s were identified for the MicA-*ompA* interactions assessed by SPR (Table 2), but these values are seen to be lower than the 190+/−32 nM K_D_ derived from earlier EMSA analysis of the interaction [26]. Such inconsistencies in the K_D_s identified for the MicA-*ompA* interaction can be explained as being a consequence of the differences in the techniques, and have been seen before for other molecular interactions. Indeed, earlier research comparing K_D_ values obtained by the two techniques for a range of different interactions have identified that SPR determined K_D_s can be between 21 and 1000 times lower, similar or even higher than those obtained by EMSA [35], [36], [37]. It is acknowledged that in the presence of slow association kinetics, as seen here for the MicA-*ompA* interaction, it may not be possible to observe stable complexes by EMSA [35]. During EMSA experiments, the duration of time spent whilst the complex associates, coupled with the period of time whilst the complex undergoes electrophoresis, may mean induced complex dissociation can occur. This would result in an observed decrease in affinity and increase in K_D_ when assessing complex formation by EMSA, as compared to that identified by SPR which monitors complex interactions under aqueous conditions in real-time. Thus, complexes need to be highly stable in order to be detected by EMSA. Nevertheless, the additional on-rate and off-rate data provided by SPR analysis of the sRNA-mRNA pair is both unique and valuable in terms of shedding light on the interactions involved [35].

The situation seen for the MicA-*ompA* interaction is not that dissimilar to what is observed for the interaction of the Qrr3 sRNA with its mRNA targets, *hapR* and *vca0939*. The Qrr3-*vca0939* interaction has a similarly slow on-rate to that observed for the MicA-*ompA* interaction, whilst the Qrr3-*hapR* on-rate is slightly faster (Table 2). However, similarly slow off-rates are observed for both Qrr3-mRNA interactions, although these are slightly faster than that for the MicA-*ompA* interaction (Table 2). The overall K_D_s identified for the Qrr3-mRNA interactions are similar to those identified for the MicA-*ompA* pairing as they are all in the low nanomolar range. However, as was seen to be the case for MicA-*ompA*, the K_D_ of the Qrr3-*hapR* interactions determined by SPR are much lower than have been identified by EMSA [15]. Specifically, EMSA analysis has identified the K_D_ of the Qrr-*hapR* interactions to be in the 250–375 nM range, whilst SPR analysis indicates a low nanomolar K_D_ for the Qrr3-*hapR* interaction. As noted above, the differences observed between the two techniques could result from it not being possible to observe stable complexes by EMSA due to the slow association and dissociation kinetics of the interaction.

Considering the slow on-rates identified for Qrr3 pairing to either *hapR* or *vca0939*, as is potentially the case for the slow on-rate seen for the MicA-*ompA* interaction, this could be due to a lack of the RNA chaperone Hfq which is known to facilitate sRNA-mRNA interactions [8]. *V. cholerae* Hfq has been shown to structurally rearrange the Qrr sRNAs, suggesting that it does so in order to promote their pairing to partner mRNAs [38]. Achieving such sRNA structural rearrangement to allow mRNA pairing in the absence of Hfq may therefore be responsible for the slow on-rate of sRNA-mRNA complex formation identified. Qrr sRNAs have, however, been shown to activate their mRNA target *vca0939* in the absence of Hfq, albeit at a lower level than when Hfq is present [14]. This illustrates that, as is the case for many sRNAs, Hfq is not strictly required for pairing but does serve to promote it [8]. For example, in *Staphylococcus aureus*, Hfq has been shown to be dispensable as Hfq-null mutants exhibit no impact on stress response, metabolic pathways or resistance to chemical agents or antibiotics, which are all response networks involving sRNAs [39]. Additionally, sRNAs have been discovered in *Mycobacterium tuberculosis* and *Helicobacter pylori*, both of which lack an Hfq homolog [40], [41], [42]. Additionally, in *V. cholerae*, the sRNA VrrA impacts its target mRNA, *ompA*, in Hfq-null mutants [43], further highlighting that some sRNAs in *V. cholerae* can function in the absence of Hfq.

Whether or not Hfq is required for sRNA-mRNA pairing *in vivo*, *in vitro* analysis of sRNA-mRNA complex off-rates can inform the stability of the pairing interaction. This provides a useful approach to allow comparison of sRNA-mRNA interactions. Whilst similar, relatively slow, off-rates have been identified for Qrr3 binding to *hapR* and *vca0939*, these are observed to be slightly faster than the off-rates identified for the MicA-*ompA* interaction. Such slow off-rates indicate stable pairing to have occurred. For mRNA targets that are down-regulated, it is possible that such stable pairing is required to provide double stranded structure to promote coupled sRNA-mRNA degradation via RNases, such as RNase III, which show preference for such double stranded character [44], [45]. In this manner, *ompA* and *hapR* could be down-regulated by MicA and the Qrr sRNAs respectively [46], [47]. By contrast, for mRNA targets that are up-regulated, stable pairing of the Qrr sRNAs to *vca0939* could be required to maintain the exposure of the formerly concealed ribosome binding site, such that ribosome binding and translation can occur [14]. Thus this approach can be seen to be a useful method for further expanding our understanding of sRNA-mRNA interactions. Indeed, by comparing the results obtained for sRNA-mRNA pairing off-rates to the values obtained for mutated sRNA variants, key nucleotides important in sRNA-mRNA pairing can be identified; this forms the basis of our future studies.

Whilst 3′ surface immobilisation of RNA is seen to be useful for unravelling interaction details within sRNA-mRNA pairing, it is not an appropriate approach for studying the direct interactions of sRNAs with the RNA chaperone protein, Hfq. Recent studies have identified the potential importance of the 3′ end of some sRNAs for binding to Hfq [48]. With the 3′ end of the RNA surface-immobilised, this region would be unavailable for binding. However, the sRNA-Hfq interaction could be studied by immobilising Hfq to the sensor surface by amine-coupling [38], [49]. Although, for investigations of the regions of sRNAs involved in binding to Hfq, blocking one binding-determinant via this means may prove useful in allowing the exploration of the other binding-determinants involved; this is also something that we are exploring. In addition, it may be possible to study the on-rate enhancement provided by Hfq through analysing the kinetics of ternary complex formation using immobilised 3′ biotin–tagged mRNA. Furthermore, the approach could prove to be useful for the study of molecular interactions with a range of other RNAs. For example, viral RNAs, which can be from several hundred nucleotides to several thousand nucleotides in length, are known to have extensive secondary and tertiary structures which confer complex regulatory roles. Such RNAs are the topic of much current research [50], [51], [52], [53]. Hence, biotin-tagging the RNAs to allow their surface immobilisation for subsequent SPR analysis of their interactions with potential binding partners could prove highly valuable and demonstrate the impact of this approach within the broader RNA-molecular interactions research field.

With their versatile functions, and the recent explosion of interest in transcriptomics, RNAs and their interactions with proteins, nucleic acids and small molecules are currently the subject of intense scientific research. RNA may represent an as yet untapped resource in the search for novel pharmaceutical drug targets [54], [55]. Our approach, therefore, has great capacity to impact both academic and industry-based research. It is expected to contribute significantly to basic research by unravelling the function of RNA-based interactions, and also to the emerging RNA-based drug discovery field.

## Supporting Information

File S1 **Includes Tables S1–S3, and Figures S1–S8.**
(PDF)

## References

[1] Cech TR (2012) The RNA worlds in context. Cold Spring Harb Perspect Biol 4: a006742. 21441585 10.1101/cshperspect.a006742 PMC3385955

[2] Serganov A, Nudler E (2013) A decade of riboswitches. Cell 152: 17–24. 23332744 10.1016/j.cell.2012.12.024 PMC4215550

[3] Kugel JF, Goodrich JA (2012) Non-coding RNAs: key regulators of mammalian transcription. Trends Biochem Sci 37: 144–151. 22300815 10.1016/j.tibs.2011.12.003 PMC3323709

[4] Fedor MJ, Williamson JR (2005) The catalytic diversity of RNAs. Nat Rev Mol Cell Biol 6: 399–412. 15956979 10.1038/nrm1647

[5] Kieft JS (2008) Viral IRES RNA structures and ribosome interactions. Trends Biochem Sci 33: 274–283. 18468443 10.1016/j.tibs.2008.04.007 PMC2706518

[6] Vogel J, Papenfort K (2006) Small non-coding RNAs and the bacterial outer membrane. Curr Opin Microbiol 9: 605–611. 17055775 10.1016/j.mib.2006.10.006

[7] Urban JH, Vogel J (2007) Translational control and target recognition by *Escherichia coli* small RNAs in vivo. Nucleic Acids Res 35: 1018–1037. 17264113 10.1093/nar/gkl1040 PMC1807950

[8] Jousselin A, Metzinger L, Felden B (2009) On the facultative requirement of the bacterial RNA chaperone, Hfq. Trends Microbiol 17: 399–405. 19733080 10.1016/j.tim.2009.06.003

[9] Altuvia S, Zhang A, Argaman L, Tiwari A, Storz G (1998) The *Escherichia coli* OxyS regulatory RNA represses *fhlA* translation by blocking ribosome binding. EMBO J 17: 6069–6075. 9774350 10.1093/emboj/17.20.6069 PMC1170933

[10] Zhang A, Altuvia S, Tiwari A, Argaman L, Hengge-Aronis R, et al (1998) The OxyS regulatory RNA represses *rpoS* translation and binds the Hfq (HF-I) protein. EMBO J 17: 6061–6068. 9774349 10.1093/emboj/17.20.6061 PMC1170932

[11] Lease RA, Cusick ME, Belfort M (1998) Riboregulation in *Escherichia coli*: DsrA RNA acts by RNA:RNA interactions at multiple loci. Proc Natl Acad Sci U S A 95: 12456–12461. 9770507 10.1073/pnas.95.21.12456 PMC22852

[12] Carpousis AJ (2003) Degradation of targeted mRNAs in *Escherichia coli*: regulation by a small antisense RNA. Genes Dev 17: 2351–2355. 14522943 10.1101/gad.1147003

[13] Lenz DH, Mok KC, Lilley BN, Kulkarni RV, Wingreen NS, et al (2004) The small RNA chaperone Hfq and multiple small RNAs control quorum sensing in *Vibrio harveyi* and *Vibrio cholerae* . Cell 118: 69–82. 15242645 10.1016/j.cell.2004.06.009

[14] Hammer BK, Bassler BL (2007) Regulatory small RNAs circumvent the conventional quorum sensing pathway in pandemic *Vibrio cholerae* . Proc Natl Acad Sci U S A 104: 11145–11149. 17556542 10.1073/pnas.0703860104 PMC1888797

[15] Bardill JP, Zhao X, Hammer BK (2011) The *Vibrio cholerae* quorum sensing response is mediated by Hfq-dependent sRNA/mRNA base pairing interactions. Mol Microbiol 80: 1381–1394. 21453446 10.1111/j.1365-2958.2011.07655.x

[16] Zhu J, Miller MB, Vance RE, Dziejman M, Bassler BL, et al (2002) Quorum-sensing regulators control virulence gene expression in *Vibrio cholerae* . Proc Natl Acad Sci U S A 99: 3129–3134. 11854465 10.1073/pnas.052694299 PMC122484

[17] Rusmini F, Zhong Z, Feijen J (2007) Protein immobilization strategies for protein biochips. Biomacromolecules 8: 1775–1789. 17444679 10.1021/bm061197b

[18] Caruthers MH (2011) A brief review of DNA and RNA chemical synthesis. Biochem Soc Trans 39: 575–580. 21428942 10.1042/BST0390575

[19] Milligan JF, Groebe DR, Witherell GW, Uhlenbeck OC (1987) Oligoribonucleotide synthesis using T7 RNA polymerase and synthetic DNA templates. Nucleic Acids Res 15: 8783–8798. 3684574 10.1093/nar/15.21.8783 PMC306405

[20] Salim NN, Feig AL (2010) An upstream Hfq binding site in the *fhlA* mRNA leader region facilitates the OxyS-*fhlA* interaction. PLoS One 5: e13028. 20927406 10.1371/journal.pone.0013028 PMC2946933

[21] Paredes E, Evans M, Das SR (2011) RNA labeling, conjugation and ligation. Methods 54: 251–259. 21354310 10.1016/j.ymeth.2011.02.008

[22] Kurschat WC, Muller J, Wombacher R, Helm M (2005) Optimizing splinted ligation of highly structured small RNAs. RNA 11: 1909–1914. 16251384 10.1261/rna.2170705 PMC1370878

[23] Richardson RW, Gumport RI (1983) Biotin and fluorescent labeling of RNA using T4 RNA ligase. Nucleic Acids Res 11: 6167–6184. 6194506 10.1093/nar/11.18.6167 PMC326365

[24] Cole K, Truong V, Barone D, McGall G (2004) Direct labeling of RNA with multiple biotins allows sensitive expression profiling of acute leukemia class predictor genes. Nucleic Acids Res 32: e86. 15205470 10.1093/nar/gnh085 PMC443553

[25] Udekwu KI, Darfeuille F, Vogel J, Reimegard J, Holmqvist E, et al (2005) Hfq-dependent regulation of OmpA synthesis is mediated by an antisense RNA. Genes Dev 19: 2355–2366. 16204185 10.1101/gad.354405 PMC1240044

[26] Henderson CA, Vincent HA, Stone CM, Phillips JO, Cary PD, et al (2013) Characterization of MicA interactions suggests a potential novel means of gene regulation by small non-coding RNAs. Nucleic Acids Res 41: 3386–3397. 23361466 10.1093/nar/gkt008 PMC3597676

[27] Andrade JM, Pobre V, Arraiano CM (2013) Small RNA modules confer different stabilities and interact differently with multiple targets. PLoS One 8: e52866. 23349691 10.1371/journal.pone.0052866 PMC3551931

[28] Gao X, Yo P, Keith A, Ragan TJ, Harris TK (2003) Thermodynamically balanced inside-out (TBIO) PCR-based gene synthesis: a novel method of primer design for high-fidelity assembly of longer gene sequences. Nucleic Acids Res 31: e143. 14602936 10.1093/nar/gng143 PMC275580

[29] Zuker M (2003) Mfold web server for nucleic acid folding and hybridization prediction. Nucleic Acids Res 31: 3406–3415. 12824337 10.1093/nar/gkg595 PMC169194

[30] Lingner J, Keller W (1993) 3′-end labeling of RNA with recombinant yeast poly(A) polymerase. Nucleic Acids Res 21: 2917–2920. 7687347 10.1093/nar/21.12.2917 PMC309683

[31] England TE, Uhlenbeck OC (1978) Enzymatic oligoribonucleotide synthesis with T4 RNA ligase. Biochemistry 17: 2069–2076. 667012 10.1021/bi00604a008

[32] Romaniuk E, McLaughlin LW, Neilson T, Romaniuk PJ (1982) The effect of acceptor oligoribonucleotide sequence on the T4 RNA ligase reaction. Eur J Biochem 125: 639–643. 7117259 10.1111/j.1432-1033.1982.tb06730.x

[33] Altuvia S, Weinstein-Fischer D, Zhang A, Postow L, Storz G (1997) A small, stable RNA induced by oxidative stress: role as a pleiotropic regulator and antimutator. Cell 90: 43–53. 9230301 10.1016/s0092-8674(00)80312-8

[34] Repoila F, Majdalani N, Gottesman S (2003) Small non-coding RNAs, co-ordinators of adaptation processes in *Escherichia coli*: the RpoS paradigm. Mol Microbiol 48: 855–861. 12753181 10.1046/j.1365-2958.2003.03454.x

[35] Matos RG, Barbas A, Arraiano CM (2010) Comparison of EMSA and SPR for the characterization of RNA-RNase II complexes. Protein J 29: 394–397. 20589527 10.1007/s10930-010-9265-1

[36] Bondeson K, Frostell-Karlsson A, Fagerstam L, Magnusson G (1993) Lactose repressor-operator DNA interactions: kinetic analysis by a surface plasmon resonance biosensor. Anal Biochem 214: 245–251. 8250230 10.1006/abio.1993.1484

[37] Henriksson-Peltola P, Sehlen W, Haggard-Ljungquist E (2007) Determination of the DNA-binding kinetics of three related but heteroimmune bacteriophage repressors using EMSA and SPR analysis. Nucleic Acids Res 35: 3181–3191. 17412705 10.1093/nar/gkm172 PMC1904268

[38] Vincent HA, Henderson CA, Stone CM, Cary PD, Gowers DM, et al (2012) The low-resolution solution structure of *Vibrio cholerae* Hfq in complex with Qrr1 sRNA. Nucleic Acids Res 40: 8698–8710. 22730296 10.1093/nar/gks582 PMC3458539

[39] Bohn C, Rigoulay C, Bouloc P (2007) No detectable effect of RNA-binding protein Hfq absence in *Staphylococcus aureus* . BMC Microbiol 7: 10. 17291347 10.1186/1471-2180-7-10 PMC1800855

[40] Li SK, Ng PK, Qin H, Lau JK, Lau JP, et al (2013) Identification of small RNAs in *Mycobacterium smegmatis* using heterologous Hfq. RNA 19: 74–84. 23169799 10.1261/rna.034116.112 PMC3527728

[41] Sharma CM, Hoffmann S, Darfeuille F, Reignier J, Findeiss S, et al (2010) The primary transcriptome of the major human pathogen *Helicobacter pylori* . Nature 464: 250–255. 20164839 10.1038/nature08756

[42] Rieder R, Reinhardt R, Sharma C, Vogel J (2012) Experimental tools to identify RNA-protein interactions in *Helicobacter pylori* . RNA Biol 9: 520–531. 22546936 10.4161/rna.20331

[43] Song T, Mika F, Lindmark B, Liu Z, Schild S, et al (2008) A new *Vibrio cholerae* sRNA modulates colonization and affects release of outer membrane vesicles. Mol Microbiol 70: 100–111. 18681937 10.1111/j.1365-2958.2008.06392.x PMC2628432

[44] Arraiano CM, Andrade JM, Domingues S, Guinote IB, Malecki M, et al (2010) The critical role of RNA processing and degradation in the control of gene expression. FEMS Microbiol Rev 34: 883–923. 20659169 10.1111/j.1574-6976.2010.00242.x

[45] Lalaouna D, Simoneau-Roy M, Lafontaine D, Masse E (2013) Regulatory RNAs and target mRNA decay in prokaryotes. Biochim Biophys Acta 1829: 742–747. 23500183 10.1016/j.bbagrm.2013.02.013

[46] Viegas SC, Silva IJ, Saramago M, Domingues S, Arraiano CM (2011) Regulation of the small regulatory RNA MicA by ribonuclease III: a target-dependent pathway. Nucleic Acids Res 39: 2918–2930. 21138960 10.1093/nar/gkq1239 PMC3074148

[47] Svenningsen SL, Waters CM, Bassler BL (2008) A negative feedback loop involving small RNAs accelerates *Vibrio cholerae’s* transition out of quorum-sensing mode. Genes Dev 22: 226–238. 18198339 10.1101/gad.1629908 PMC2192756

[48] Sauer E, Weichenrieder O (2011) Structural basis for RNA 3′-end recognition by Hfq. Proc Natl Acad Sci U S A 108: 13065–13070. 21737752 10.1073/pnas.1103420108 PMC3156190

[49] Henderson CA, Vincent HA, Casamento A, Stone CM, Phillips JO, et al (2013) Hfq binding changes the structure of *Escherichia coli* small noncoding RNAs OxyS and RprA, which are involved in the riboregulation of *rpoS* . RNA 19: 1089–1104. 23804244 10.1261/rna.034595.112 PMC3708529

[50] Alvarez DE, Lodeiro MF, Luduena SJ, Pietrasanta LI, Gamarnik AV (2005) Long-range RNA-RNA interactions circularize the dengue virus genome. J Virol 79: 6631–6643. 15890901 10.1128/JVI.79.11.6631-6643.2005 PMC1112138

[51] Andersen ES, Contera SA, Knudsen B, Damgaard CK, Besenbacher F, et al (2004) Role of the trans-activation response element in dimerization of HIV-1 RNA. J Biol Chem 279: 22243–22249. 15014074 10.1074/jbc.M314326200

[52] Shetty S, Stefanovic S, Mihailescu MR (2013) Hepatitis C virus RNA: molecular switches mediated by long-range RNA-RNA interactions? Nucleic Acids Res 41: 2526–2540. 23275555 10.1093/nar/gks1318 PMC3575821

[53] Friebe P, Pena J, Pohl MO, Harris E (2012) Composition of the sequence downstream of the dengue virus 5′ cyclization sequence (dCS) affects viral RNA replication. Virology 422: 346–356. 22137186 10.1016/j.virol.2011.10.025 PMC3607303

[54] Vicens Q, Westhof E (2003) RNA as a drug target: the case of aminoglycosides. Chembiochem 4: 1018–1023. 14523919 10.1002/cbic.200300684

[55] Vicens Q (2009) RNA’s coming of age as a drug target. Journal of Inclusion Phenomena and Macrocyclic Chemistry 65: 171–188.

