# Supplementary material for: An Improved Method for Surface Immobilisation of RNA: Application to Small Non-Coding RNA - mRNA Pairing
Source: PLoS One. 2013 Nov 14;8(11):e79142. doi: 10.1371/journal.pone.0079142 (PMC3828260; doi:10.1371/journal.pone.0079142)

Supporting Information for Manuscript:

An improved method for surface immobilisation of RNA: application to small non-coding RNA - mRNA pairing.

Helen A. Vincent, Jack O. Phillips, Charlotte A. Henderson, Adam J. Roberts, Carlanne M. Stone, Charlotte E. Mardle, Louise E. Butt, Darren M. Gowers, Andrew R. Pickford, and Anastasia J. Callaghan

TABLES

Table S1. RNA sequence details. The poly-A-tails are highlighted in bold. For each mRNA the start codon (not present in *rpoS* (short)) is underlined.

RNA name	Number of nucleotides	RNA sequence with poly-A-tails highlighted in bold
sRNA Qrr1	99	GGGUGACCCGCAAGGGUCACCUAGCCAACUGACGUUGUUAGUGAAUAAUCA<br>AUGUUCACAAAUAACAGCCAAUAGACUCAUUCUAUUGGCU**AAAAAAAA**#<br>#**A**-tail was also replaced with equivalent C's and U's for testing
sRNA Qrr2	110	GGGUGACCCUUGUUAAGCCGAGGGUCACCUAGCCAACUGACGUUGUUAGUG<br>AAUAGUAUUGUUCACAUCAUAUAUAAGCCAUCGCGGUUCUUGCGAUUGGCU<br>**AAAAAAAA**
sRNA Qrr3	110	GGGUGACCCUUAAUUAAGCCGAGGGUCACCUAGCCAACUGACGUUGUUAGU<br>GAAUGAAAUUGUUCACAUUUGUUUUAUCAGCCAAUCACCCUUUUGUGAUUGG<br>C**AAAAAA**
sRNA Qrr4	110	GGGUGACCCUUCUAAGCCGAGGGUCACCUAGCCAACUGACGUUGUUAGUGA<br>ACACCAUUGUUCACACUUAUAGACGGCCAAUCACACUUCUUGUGGUUGGCC<br>**AAAAAAAA**
hapR mRNA position -80 to +21	101	GGGCUUUAAGUAGCAAAUAACAAAAUAAUCAUUAGAGCAAAAUGCUCAAUCAA<br>CAACUCAAUUGGCAAGGAUAUACCCCUAUGGACGCAUCAAUCG**AAAAA**
vca0939 mRNA position -50 to +8	61	GGGAGGCGUUUAAAAAUAACGAUUGGCUAGGUUCCCCAAGCCCGAAGCAAC<br>CGAUG**AAAAA**
rpoS mRNA (short) position -576 to -172	413	GGGUUCUGAGUCUUCGGGUGAACAGAGUGCUAACAAAAUGUUGCCGAACAA<br>CAAGCCAACUGCGACCACGGUCACAGCGCCUGUAACGGUACCAACAGCAAGC<br>ACAACCGAGCCGACUGUCAGCAGUACAUCAACCAGUACGCCUAUCUCCACCU<br>GGCGCUGGCCGACUGAGGGCAAAGUGAUCGAAACCUUUGGCGCUUCUGAGG<br>GGGGCAACAAGGGGAUUGAUAUCGCAGGCAGCAAAGGACAGGCAAUUAUCG<br>CGACCGCAGAUGGCCGCGUUGUUUAUGCUGGUAACGCGCUGCGCGGCUAC<br>GGUAAUCUGAUUAUCAUCAAACAUAAUGAUGAUUACCUGAGUGCCUACGCCC<br>AUAACGACACAAUGCUGGUCCGGGAACAACAAGAAGUUAAGGCGGGGC**AAA**<br>**AAA**
sRNA MicA	79	GGGGAAAGACGCGCAUUUGUUAUCAUCAUCCCUGAAUUCAGAGAUGAAAUUU<br>UGGCCACUCACGAGUGGCCUUUU**AAAA**#<br>#**A**-tail was also replaced with equivalent G's and U's for testing<br>#**A**-tails of 0-3 nucleotides in length were also tested
ompA mRNA position -128 to +32	164	GGGUGCUCGGCAUAAGCCGAAGAUAUCGGUAGAGUUAAUAUUGAGCAGAUC<br>CCCCGGUGAAGGAUUUAACCGUGUUAUCUCGUUGGAGAUAUUCAUGGCGUA<br>UUUUGGAUGAUAACGAGGCGCAAAAAAUGAAAAAGACAGCUAUCGCGAUUGC<br>AGUGGC**AAAA**
rpoS mRNA (long) position -576 to +9	592	GGGUUCUGAGUCUUCGGGUGAACAGAGUGCUAACAAAAUGUUGCCGAACAA<br>CAAGCCAACUGCGACCACGGUCACAGCGCCUGUAACGGUACCAACAGCAAGC<br>ACAACCGAGCCGACUGUCAGCAGUACAUCAACCAGUACGCCUAUCUCCACCU<br>GGCGCUGGCCGACUGAGGGCAAAGUGAUCGAAACCUUUGGCGCUUCUGAGG<br>GGGGCAACAAGGGGAUUGAUAUCGCAGGCAGCAAAGGACAGGCAAUUAUCG<br>CGACCGCAGAUGGCCGCGUUGUUUAUGCUGGUAACGCGCUGCGCGGCUAC<br>GGUAAUCUGAUUAUCAUCAAACAUAAUGAUGAUUACCUGAGUGCCUACGCCC<br>AUAACGACACAAUGCUGGUCCGGGAACAACAAGAAGUUAAGGCGGGGCAAAA<br>AAUAGCGACCAUGGGUAGCACCGGAACCAGUUCAACACGCUUGCAUUUUGAA<br>AUUCGUUACAAGGGGAAAUCCGUAAACCCGCUGCGUUAUUUGCCGCAGCGA<br>UAAAUCGGCGGAACCAGGCUUUUGCUUGAAUGUUCCGUCAAGGGAUCACGG<br>GUAGGAGCCACCUUAUGAGUCAG**AAAA**
sRNA DsrA	94	GGGAACACAUCAGAUUUCCUGGUGUAACGAAUUUUUUAAGUGCUUCUUGCU<br>UAAGCAAGUUUCAUCCCGACCCCCUCAGGGUCGGGAUUU**AAAA**

Table S2. DNA primer sequences for preparing RNAs with and without 3' poly-A-tails. The +As reverse primer (labelled) substitutes for the outside reverse primer when making the poly-A-tail DNA template.

RNA name	Primer name	DNA sequence (5' to 3') with poly-A-tails in bold
sRNA Qrr1	Sense 2	TAATACGACTCACTATAGGGTGACCCGCAAGGGTCAC
	Sense 1	GTGACCCGCAAGGGTCACCTAGCCAACTGACGTTGTTAGTGAATAATC
	Antisense 1	GAATGAGTCTATTGGCTGTTATTTGTGAACATTGATTATTCACTAACAACGTCAGTTGGC
	Antisense 2	AAAAAAATAGCCAATAGAATGAGTCTATTGGCTGTTATTTGTGAAC
	Antisense 2.2 (+As reverse primer)	#**TTTTTTTT**AGCCAATAGAATGAGTCTATTGGCTGTTATTTGTGAAC<br>#**T**'s were replaced with G's and A's for *in vitro* transcription of RNAs with poly-C and poly-U tails respectively.
sRNA Qrr2	Sense 2	TAATACGACTCACTATAGGGTGACCCTTGTTAAGCCGAG
	Sense 1	TGACCCGCAAGGGTCACCTAGCCAACTGACGTTGTTAGTGAATAATC
	Antisense 1	CGCGATTGGCTTATATATGATGTGAACAATACTATTCACTAACAACGTCAGTTGGCTAGG
	Antisense 2	AAAAAAATAGCCAATCGCAAGAACCGCGATTGGCTTATATATGATGTGAAC
	Antisense 2.2 (+As reverse primer)	**TTTTTTTT**AGCCAATCGCAAGAACCGCGATTGGCTTATATATGATGTGAAC
sRNA Qrr3	Sense 2	TAATACGACTCACTATAGGGTGACCCTTAATTAAGCCGAG
	Sense 1	GGGTGACCCTTAATTAAGCCGAGGGTCACCTAGCCAACTGACGTTGTTAG
	Antisense 1	GATTGGCTGATAAAACAAATGTGAACAATTTCATTCACTAACAACGTCAGTTGGCTAGGTG
	Antisense 2	AAAAAAGCCAATCACAAAAGGGTGATTGGCTGATAAAACAAATGTGAACAATTTCATTC
	Antisense 2.2 (+As reverse primer)	**TTTTTT**GCCAATCACAAAAGGGTGATTGGCTGATAAAACAAATGTGAACAATTTCATTC
sRNA Qrr4	Sense 2	TAATACGACTCACTATAGGGTGACCCTTCTAAGCCGAGG
	Sense 1	GTGACCCTTCTAAGCCGAGGGTCACCTAGCCAACTGACGTTGTTAGTGAACACC
	Antisense 1	GTGTGATTGGCCGTCTATAAGTGTGAACAATGGTGTTCACTAACAACGTCAGTTGG
	Antisense 2	AAAAAAAAGGCCAACCACAAGAAGTGTGATTGGCCGTCTATAAGTGTG
	Antisense 2.2 (+As reverse primer)	**TTTTTTTT**GGCCAACCACAAGAAGTGTGATTGGCCGTCTATAAGTGTG
mRNA 5'UTR *hapR*	Sense 2	TAATACGACTCACTATAGGGCTTTAAGTAGCAAATAACAAAATAATCATTAGAG
	Sense 1	GGGCTTTAAGTAGCAAATAACAAAATAATCATTAGAGCAAAATGCTCAATCAACAACTC
	Antisense 1	GGGGTATATCCTTGCCAATTGAGTTGTTGATTGAGCATTTTGCTCTAATG
	Antisense 2	ATGCGTCCATAGGGGTATATCCTTGCCAATTGAG
	Antisense 2.2 (+As reverse primer)	**TTTTT**CGATTGATGCGTCCATAGGGGTATATCCTTGCCAATTGAG
mRNA 5'UTR *vca0939*	Sense	TAATACGACTCACTATAGGGAGGCGTTTAAAAATAACGATTGGCTAGGTTCCCCAAG
	Antisense (+As reverse primer)	**TTTTT**CATCGGTTGCTTCGGGCTTGGGGAACCTAGCCAATCG
mRNA *rpoS* (short)	Sense	TAATACGACTCACTATAGGGTTCTGAGTCTTCGGGTGAACAG
	Antisense (+As reverse primer)	**TTTTTT**GCCCCGCCTTAACTTCTTG
sRNA MicA	Sense	TAATACGACTCACTATAGGGGAAAGACGCGCATTTGTTATCATCATCCCTGAATTCAGAG
	Antisense	AAAAGGCCACTCGTGAGTGGCCAAAATTTCATCTCTGAATTCAGGGATGATGATAA
	Antisense (+As reverse primer)	#**TTTT**AAAAGGCCACTCGTGAGTGGCCAAAATTTCATCTCTGAATTCAGGGATGATGATAA<br>#**T**'s were replaced with C's and A's for *in vitro* transcription of RNAs with poly-G and poly-U tails respectively.<br>#the number of T's was amended depending on the length of the poly-A-tail tested
mRNA 5'UTR *ompA*	Sense 3	TAATACGACTCACTATAGGGCCAGGGGT
	Sense 2	CTCACTATAGGGCCAGGGGTGCTCGGCATAAGCCGAAGATATCGGTAGAGTTAATATTGAGC
	Sense 1	CCGAAGATATCGGTAGAGTTAATATTGAGCAGATCCCCCGGTGAAGGATTTAACCGTG

	Antisense 1	CCAAAATACGCCATGAATATCTCCAACGAGATAACACGGTTAAATCCTTCACCGGG
	Antisense 2	GTCTTTTTCATTTTTTGCGCCTCGTTATCATCCAAAATACGCCATGAATATCTCCAACG
	Antisense 3	TGCCACTGCAATCGCGATAGCTGTCTTTTTCATTTTTTGCGCCTCGTTATC
	Antisense 3.2 (+As reverse primer)	**TTTT**GCCACTGCAATCGCGATAGCTGTCTTTTTCATTTTTTGCGCCTCGTTATC
mRNA *rpoS* (long)	Sense	TAATACGACTCACTATAGGGTTCTGAGTCTTCGGGTGAACAG
	Antisense	TCTGACTCATAAGGTGGCTCC
	Antisense (+As reverse primer)	**TTTT**CTGACTCATAAGGTGGCTCC
sRNA DsrA	Sense	TAATACGACTCACTATAGGGAACACATCAGATTTCCTGGTGTAACGAATTTTTTAAGTGCTTCTTGC
	Antisense	AAATCCCGACCCTGAGGGGGTCGGGATGAAACTTGCTTAAGCAAGAAGCACTTAAAAAATTCGTTAC
	Antisense (+As reverse primer)	**TTTT**AAATCCCGACCCTGAGGGGGTCGGGATGAAACTTGCTTAAGCAAGAAGCACTTAAAAAATTCGTTAC

Table S3. Ligation yields for ligation of U_5-biotin to RNAs incorporating poly-A-tails. The sequences of the RNAs are given in Table S1. Yields were calculated as described in Materials and Methods.

**RNA**	sRNA Qrr1	sRNA Qrr2	sRNA Qrr3	sRNA Qrr4	*hapR* mRNA	*vca0939* mRNA	sRNA MicA	*ompA* mRNA	sRNA DsrA
**% Yield**	31	27	39	43	41	40	26	29	34

FIGURES

Figure S1. Chemical structure of U-biotin.

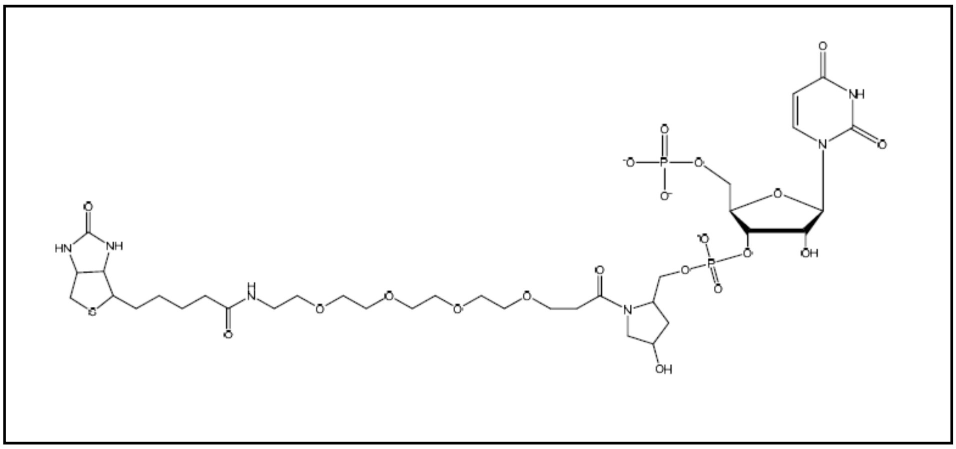

Figure S2. Comparison of U-biotin ligation to RNAs with and without poly-A-tails. Analysis of the ligation reactions for a) Qrr3 and b) DsrA with and without poly-A-tails. Gels were stained with SYBR-Gold and blots were probed with streptavidin-HRP to detect biotin-labelled RNA. Schematic representations of RNA species identified on the gels/blots are shown. The sequences of the RNAs are given in Table S1.

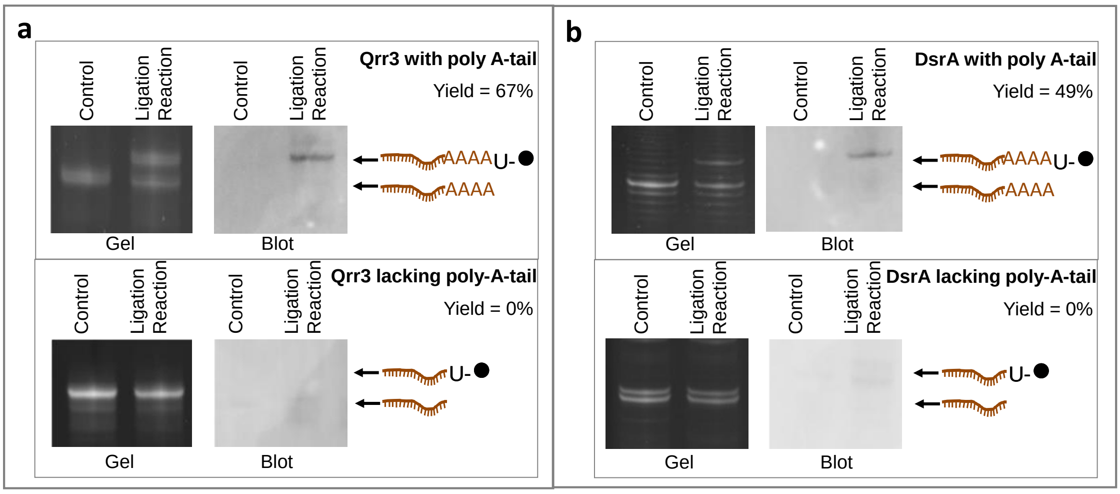

Figure S3. Ligation of U-biotin to RNAs with poly-A-tails. Analysis of the ligation reaction for (a) Qrr1, (b) Qrr4 and (c) MicA with poly-A-tails. Gels were stained with SYBR-Gold and blots were probed with streptavidin-HRP to detect biotin-labelled RNA. Schematic representations of RNA species identified on the gels/blots are shown. The sequences of the RNAs are given in Table S1.

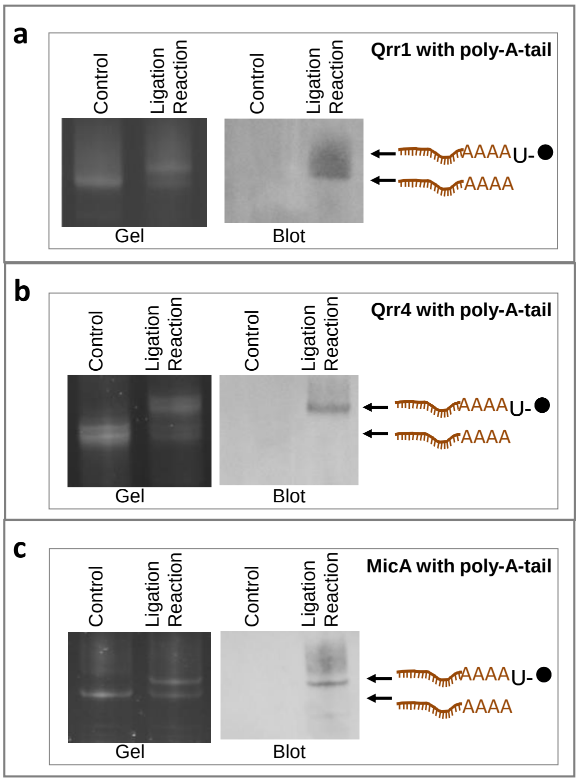

Figure S4. Spin column removal of U-biotin is effective. Chromatograms of analytical gel filtration runs of samples on a Zorbax 450 column (Agilent), each shown normalised to the largest peak. MicroBioSpin 6 columns (Bio-Rad) were used (according to the manufacturers protocol) to separate the excess U-biotin reagent from the RNA. At each stage, samples were removed and analysed by size exclusion chromatography to prove the effectiveness of the method. The top panels show the data for the first stage, that of the ligation reaction mixture which was loaded onto the MicroBioSpin column. It is seen to contain both RNA and the ligation reaction reagent. The middle panels show the data for the second stage, when the RNA is eluted from the MicroBioSpin column. At this point, the samples are seen only to contain RNA. Finally, the bottom panels show the data for the last stage, the samples retained by the MicroBioSpin column are tested and shown to contain the ligation reaction reagents. The data is shown for the RNAs, a) DsrA, b) MicA, c) *ompA* and d) Qrr2. The sequences of the RNAs tested are given in Table S1.

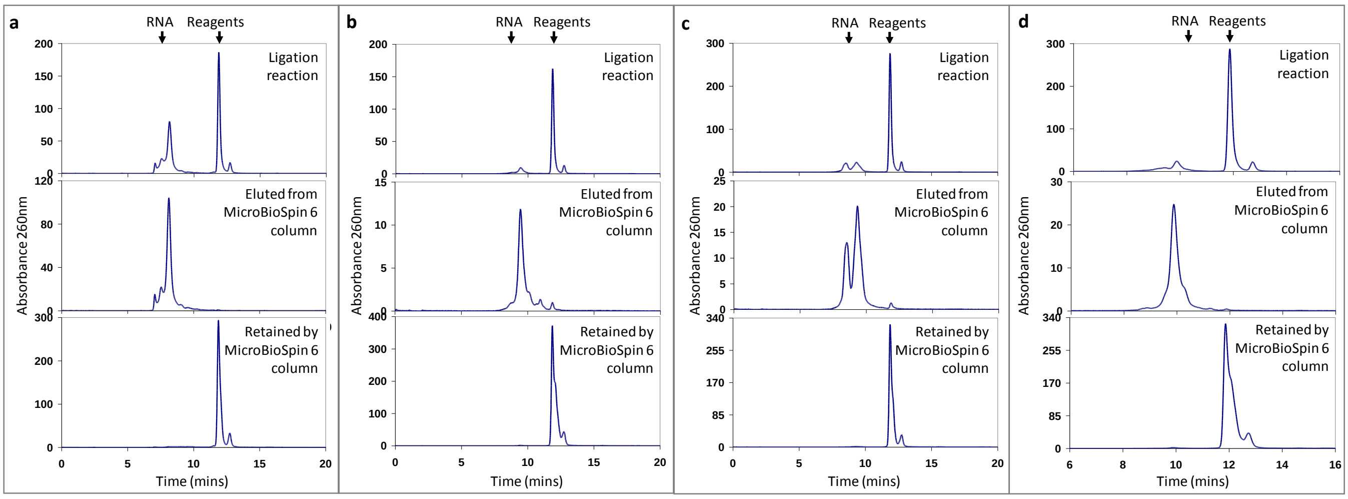

Figure S5. Immobilisation of biotin-labelled RNA to a streptavidin-coated SPR sensor chip. Injection of a) biotin-labelled RNA and b) non- biotin-labelled RNA. Inject start and end positions are labelled. An increase in RUs is seen for the biotin-labelled RNA, illustrating binding to streptavidin to be occurring and immobilisation of the RNA to the surface of the sensor chip.

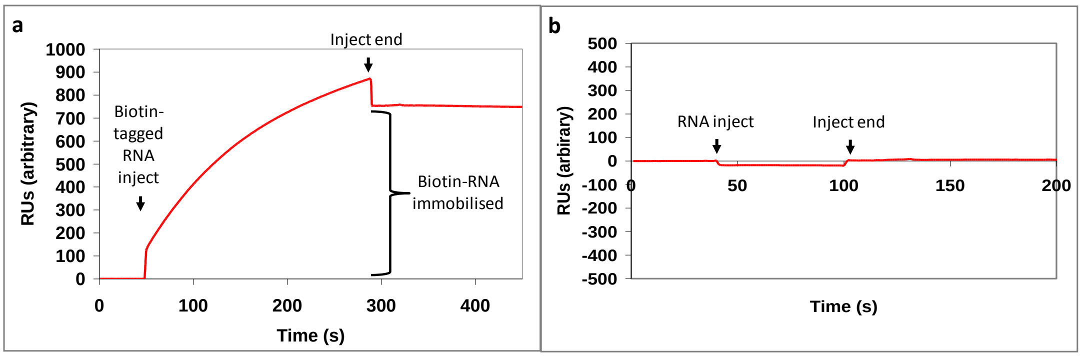

Figure S6. 3'-tail nucleotide preference. Preliminary data indicates poly-A-tail is the preferred 3' end. Panel 1) Ligation and MFold analysis of MicA with differing 3' tails: a) MFold analysis of MicA, provided as a comparison to the predicted structures of MicA with poly-U, -G, -A and -C tails (b-e). b) MFold analysis of MicA with a poly-U-tail with corresponding ligation reaction gel. c) and d) as for b) but for MicA with poly-G and poly-A tails respectively. e) MFold analysis of MicA with a poly-C-tail indicating the predicted structure to be disrupted from the native form and therefore was not tested experimentally. Ligation was only observed for MicA with a poly-A-tail (d). Panel 2) As for MicA, but for Qrr1. In this case, Qrr1 with a poly-G-tail (e) was found to disrupt the native predicted MFold structure (a) and was therefore not tested experimentally. Please note that in Panel 2 (c) the Qrr1 (poly-C-tail) RNA contains an impurity which is visible on the gel in both the control and ligation reaction samples, but it is still possible to observe that no ligation product is generated in the ligation reaction. Ligation was observed for Qrr1 with both poly-A (d) and poly-U (b) tails, but was noticeably more effective for the poly-A tail (d). Work subsequently proceeded using only poly-A-tails. The RNAs incorporating poly-C, -U, or -G tails were synthesised using the same method as that used for synthesising RNAs incorporating poly-A-tails (as described in the Materials and Methods section). The DNA primer sequences used are provided in Table S2.

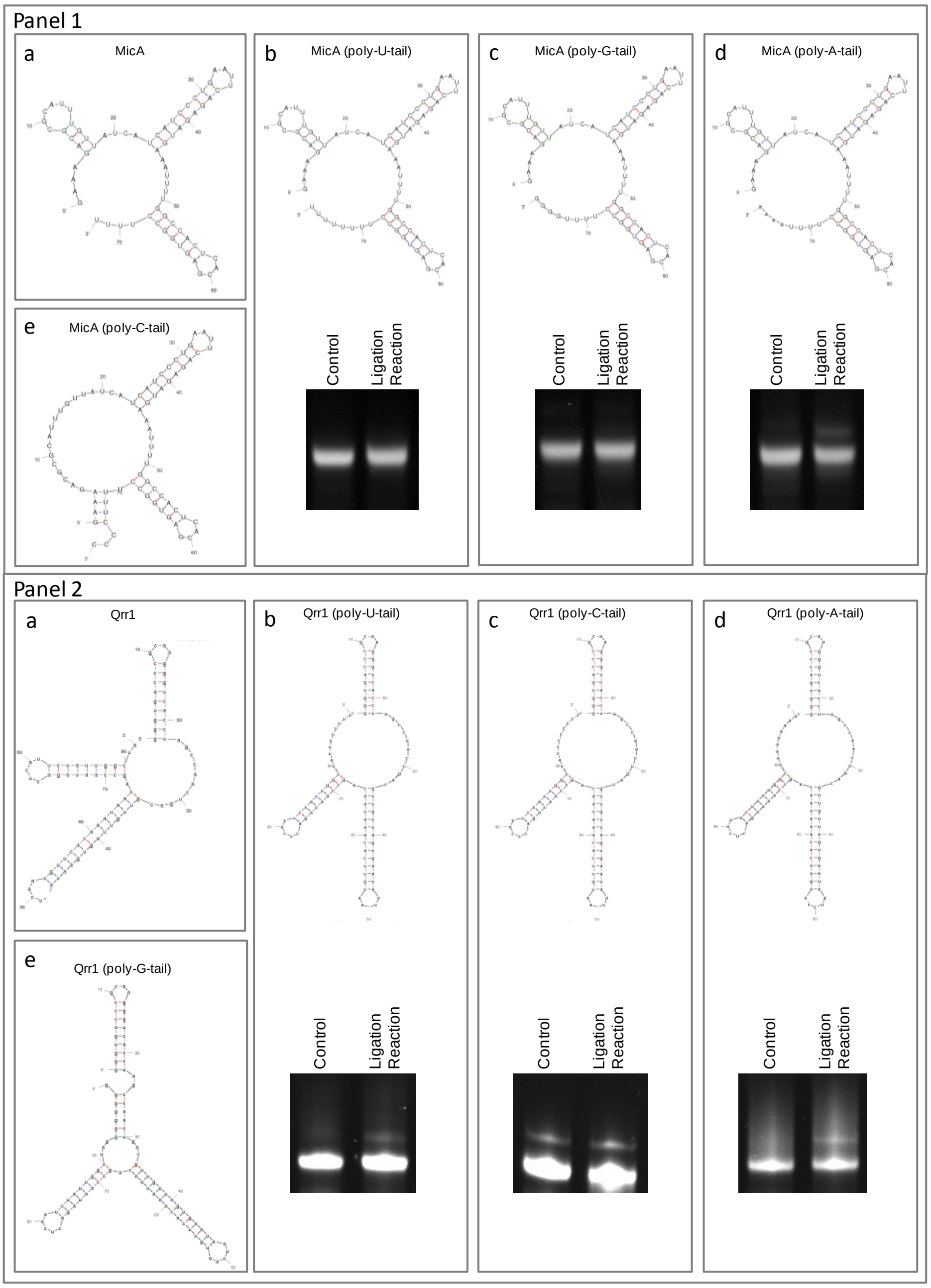

Figure S7. 3' end A-tail length impacts on ligation efficiency. Preliminary analysis of the ligation reactions for MicA with increasing A-tail length. Data are shown for MicA with 0, 1, 2 and 3, additional adenine nucleotides appended to the 3' end, as noted beneath each gel. Ligation of U-biotin is only seen for MicA with a minimum poly-A-tail of 3 nucleotides in length. The RNAs were synthesised as described in the Materials and Methods section. The DNA primer sequences used are provided in Table S2.

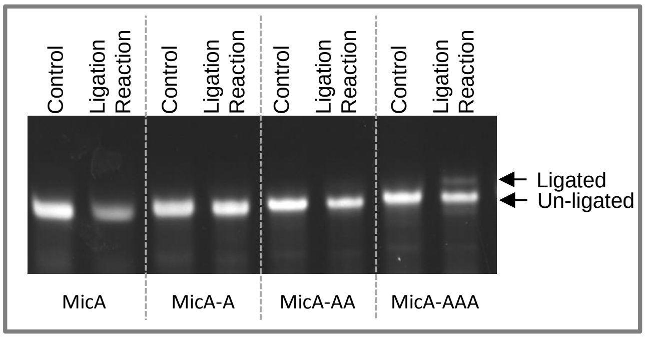

Figure S8. Ligation of U_5-biotin to RNAs. Analysis of the ligation reaction for a) Qrr2, and b) Qrr3 with and without poly-A-tails. Gels were stained with SYBR-Gold. Schematic representations of RNA species identified on the gels are shown. The sequences of the RNAs are given in Table S1.

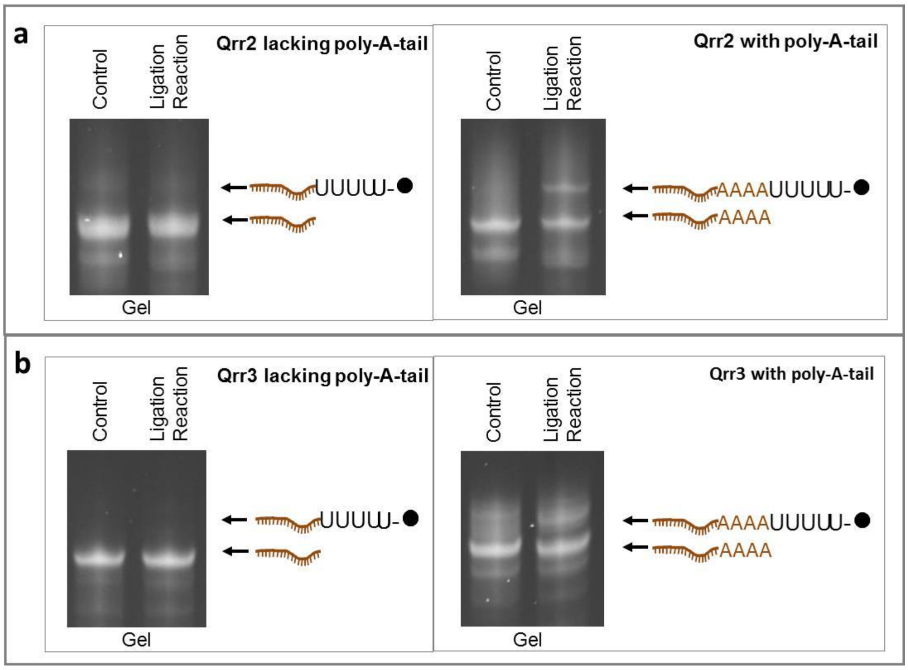

Supplement: File S1 — Includes Tables S1–S3, and Figures S1–S8. (PDF) [file pone.0079142.s001.pdf]
